# Is the Efficacy of Adding Ramucirumab to Docetaxel Related to a History of Immune Checkpoint Inhibitors in the Real-World Clinical Practice?

**DOI:** 10.3390/cancers14122970

**Published:** 2022-06-16

**Authors:** Tadashi Nishimura, Hajime Fujimoto, Tomohito Okano, Masahiro Naito, Chikashi Tsuji, Soichi Iwanaka, Yasumasa Sakakura, Taro Yasuma, Corina N. D’Alessandro-Gabazza, Yasuhiro Oomoto, Esteban C. Gabazza, Tetsu Kobayashi, Hidenori Ibata

**Affiliations:** 1 Department of Pulmonary Medicine, Mie Chuo Medical Center, Tsu City 514-1101, Japan; nishimura.tadashi.tx@mail.hosp.go.jp (T.N.); naito.masahiro.jw@mail.hosp.go.jp (M.N.); tusji.chikashi.hc@mail.hosp.go.jp (C.T.); iwanaka.soichi.zd@mail.hosp.go.jp (S.I.); sakakura.yasumasa.sy@mail.hosp.go.jp (Y.S.); oomoto.yasuhiro.gd@mail.hosp.go.jp (Y.O.); ibatah@miechuo-m.hosp.go.jp (H.I.); 2 Department of Pulmonary and Critical Care Medicine, Graduate School of Medicine, Mie University, Tsu City 514-8507, Japan; fjmt1974@clin.medic.mie-u.ac.jp (H.F.); okatomojin525@med.mie-u.ac.jp (T.O.); ktetsu@clin.medic.mie-u.ac.jp (T.K.); 3 Department of Immunology, Graduate School of Medicine, Mie University, Tsu City 514-8507, Japan; t-yasuma0630@clin.medic.mie-u.ac.jp (T.Y.); dalessac@clin.medic.mie-u.ac.jp (C.N.D.-G.)

**Keywords:** non-small cell lung cancer, immune checkpoint inhibitor, ramucirumab, docetaxel, vascular endothelial growth factor

## Abstract

**Simple Summary:**

Previous studies have shown that the use of chemotherapy in combination with immune checkpoint inhibitors as a first-line treatment in patients with non-small cell lung cancer improved overall survival and progression-free survival. However, the efficacy of cytotoxic agents as a second-line or later-line therapy in non-small cell lung cancer patients previously treated with immune checkpoint inhibitors in the real-world clinical practice is still controversial. In the present study, we retrospectively evaluated patients with non-small cell lung cancer to clarify whether the previous treatment with immune checkpoint inhibitors impacts the efficacy of docetaxel or the combined therapy of docetaxel plus ramucirumab. The results of this study using real-world data show that the addition of ramucirumab to docetaxel is superior to docetaxel monotherapy for improving time-to-treatment failure and overall survival, irrespective of previous treatment with immune checkpoint inhibitors.

**Abstract:**

Reports on the efficacy of second-line treatment with cytotoxic agents after treatment with immune checkpoint inhibitors are limited. Here, we retrospectively evaluated patients in the real-world clinical practice treated with docetaxel or docetaxel plus ramucirumab. Ninety-three patients treated with docetaxel or docetaxel plus ramucirumab as a second- or later-line therapy were included. The patients were categorized into the following four treatment groups: docetaxel group (*n* = 50), docetaxel/ramucirumab group (*n* = 43) and pretreated (*n* = 45) and untreated (*n* = 48) with immune checkpoint inhibitor groups. The docetaxel/ramucirumab group showed an overall response rate of 57.1% in patients pretreated with immune checkpoint inhibitors and 20% in untreated patients. The docetaxel group showed an overall response rate of 15.4% in patients pretreated with immune checkpoint inhibitors and 5.0% in untreated patients. The median time-to-treatment failure and the median survival time were longer in the docetaxel/ramucirumab group than in the docetaxel group in both immune checkpoint inhibitor-pretreated and -untreated groups. There was no difference in time-to-treatment failure and overall survival between immune checkpoint inhibitor-pretreated and -untreated groups in each docetaxel and docetaxel/ramucirumab treatment group. In conclusion, our real-world data show that the addition of ramucirumab to docetaxel was superior to docetaxel monotherapy for improving time-to-treatment failure and overall survival, irrespective of previous treatment with immune checkpoint inhibitors.

## 1. Introduction

There are a variety of treatment options for non-small cell lung cancer. The first-line treatment with chemotherapy in combination with immune checkpoint inhibitors (ICIs) has shown good results in terms of overall survival (OS) and progression-free survival (PFS) [1,2,3,4,5,6]. A few clinical trials have demonstrated the efficacy of second-line treatment with cytotoxic agents in patients previously treated with ICIs. Another reported therapeutic option is the combination (DTX/RAM) of ramucirumab (RAM), an anti-vascular endothelial growth factor receptor (VEGFR) antibody, with docetaxel (DTX) [7]. The comparative clinical trial of DTX/RAM versus placebo/DTX or the REVEL trial showed survival benefits as a second-line treatment of stage IV non-small cell lung cancer (NSCLC) after disease progression on platinum-based therapy [7]. Recent studies have also revealed the efficacy of the DTX/RAM combination therapy in patients with a history of ICI treatment [8,9,10,11,12,13,14]. Although the combination of chemotherapy and ICIs is currently becoming the standard regimen as the first-line treatment of advanced NSCLC, there is an insufficient number of studies regarding the use of chemotherapy and ICI combination as a second-line or later-line therapy. In this study, we retrospectively evaluated the clinical efficacy of DTX or DTX/RAM as a second-line or later-line treatment in patients with or without previous history of ICI treatment using data from the real world.

## 2. Materials and Methods

### 2.1. Patients and Study Design

**Figure 1:**
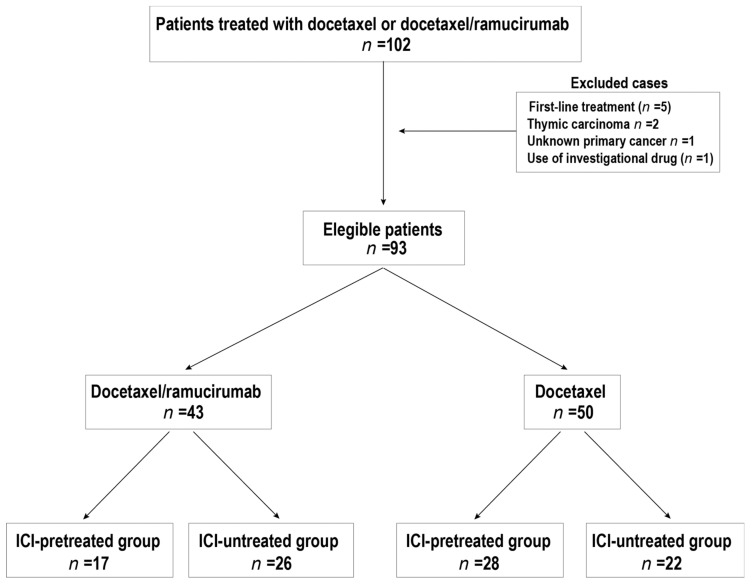
Study flow chart. The patients were divided into the DTX and DTX/RAM treatment groups. DTX: docetaxel. ICI: Immune checkpoint inhibitor. RAM ramucirumab.

This study included 102 patients treated in our institution with DTX or DTX/RAM as a second-line or later-line therapy between June 2016 and October 2021 (Figure 1). The dose of DTX in both arms was 60 mg/m^2^ every three weeks, whereas the dose of RAM in the DTX/RAM arm was 10 mg/kg every three weeks. DTX monotherapy, but not DTX/RAM combination, was indicated in the elderly, in patients with poor performance status, in tumors located near large vessels or the trachea, or in tumors that form cavitations (squamous cell carcinoma). RAM was not indicated in these cases because of the risk of bleeding. The response was not evaluated in a relatively high number of patients treated with combination therapy (*n* = 9) because the treatment was completed without performing a CT study.

Exclusion criteria were the use of DTX in non-lung cancer or as first-line therapy and the use of investigational drugs (Figure 1). The patients were categorized into DTX (*n* = 50) and DTX/RAM (*n* = 43) treatment groups. These treatment groups were also categorized into an ICI-pretreated group (*n* = 45) and an ICI-untreated group (*n* = 48).

### 2.2. Ethical Statement

The Committee for Clinical Investigation of Mie Chuo Medical Center approved the protocol of the current clinical investigation (Approval No. MCERB-202141; approval date: 12 December 2021).

### 2.3. Statistical Analysis

The Response Evaluation Criteria in Solid Tumors (RECIST) version 1.1 was used to determine the overall response rate (ORR) and the disease control rate (DCR). The time-to-treatment failure (TTF) and OS were assessed using the Kaplan-Meier curve and log-rank test. Categorical variables were evaluated using the Fisher’s test and multivariate analysis using the Cox proportional hazards regression. The hazard ratios were also calculated after adjusting for confounding factors including age, gender, smoking status, histology, driver mutation, PD-L1 status, and lung honeycombing with inverse probability of treatment weighting (IPTW) using propensity scores as previously described [14].

A *p* < 0.05 was considered significant. The statistical analysis was performed using the R software package version 4.0.3 (R Development Core Team, Vienna, Austria) and the EZR version 1.54 (Saitama Medical Center, Jichi Medical University, Saitama, Japan) [15].

## 3. Results

### 3.1. Patient Characteristics

The characteristics of the patients are shown in Table 1. Among 93 patients who fulfilled the study’s criteria, 50 patients were treated with DTX and 43 with DTX/RAM as a second-line or later-line therapy. In addition, 45 of the 93 patients were in the ICI-pretreated group, and 48 patients were in the ICI-untreated group (Figure 1). There were more cases of squamous cell carcinoma in the monotherapy (DTX) group and adenocarcinoma in the DTX/RAM group because RAM was not indicated in patients with squamous cell carcinoma due to the risk of bleeding. Squamous cell carcinoma is more commonly associated with smoking; therefore, the number of smokers was also higher in patients receiving monotherapy than in cases treated with the DTX/RAM combination.

### 3.2. Tumor Overall Response Rate and the Disease Control Rate

We first compared the tumor overall response rate and disease control rate between patients treated with DTX and DTX/RAM. The overall response rate was 10.9% in the DTX group and 35.3% in the DTX/RAM group, whereas the disease control rate was 30.4% in the DTX group and 73.5% in the DTX/RAM group (Table 2).

We then compared the tumor overall response rate and disease control rate between patients with (ICI-pretreated group) and without (ICI-untreated group) previous ICI treatment within each DTX-treated and DTX/RAM-treated group. Among patients treated with DTX/RMA, the tumor objective response rate was 57.1% in the ICI-pretreated group compared to 20% in the ICI-untreated group, while the disease control rate was 71.4% in the ICI-pretreated group compared to 75.0% in the ICI-untreated group. These results show that the disease control rate remains unchanged and that the tumor objective response rate was high in patients pretreated with ICI in both the DTX and DTX/RAM groups (Table 2).

### 3.3. Survival Analysis

**Figure 2:**
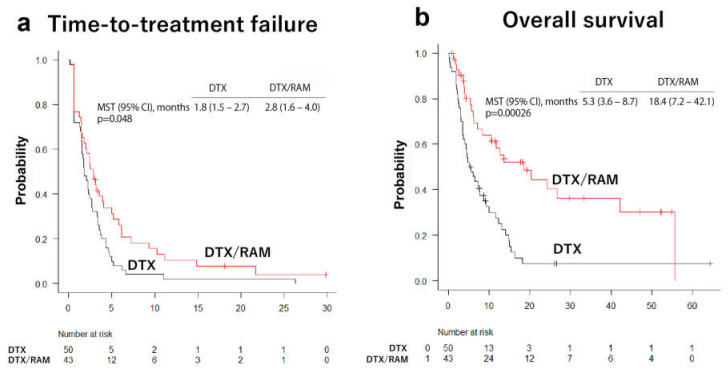
The time-to-treatment failure and overall survival in all patients. The overall survival was significantly improved by the combined treatment with docetaxel and ramucirumab (DTX/RAM) compared to docetaxel (DTX) therapy alone. No difference was observed in time-to-treatment failure between both treatment groups. MST, median survival time.

**Figure 3:**
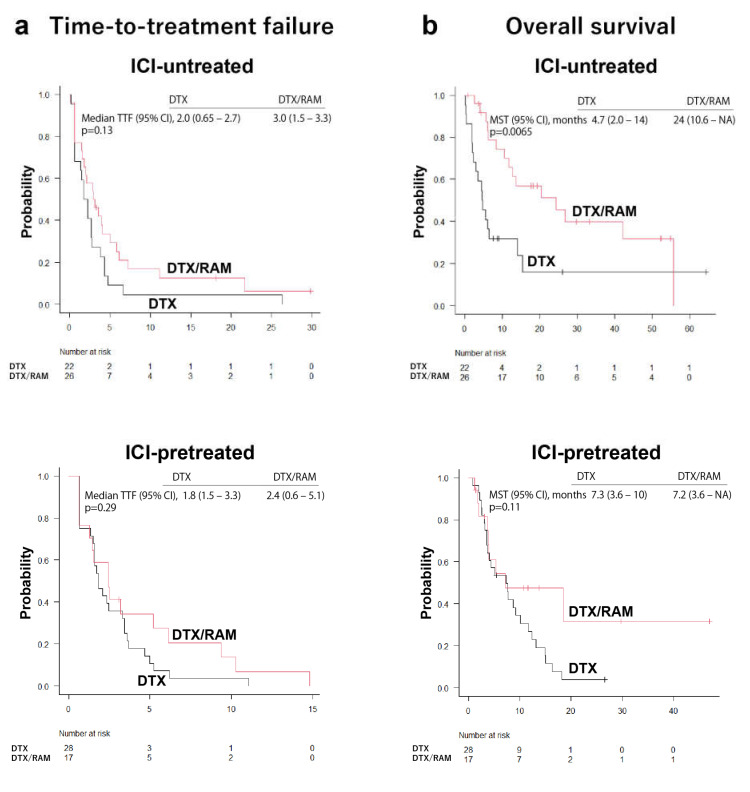
Comparative analysis of docetaxel alone and combination therapy of docetaxel and ramucirumab in all immune checkpoint inhibitor-pretreated and -untreated patients. The time-to-treatment failure (TTF) was not significantly different between docetaxel (DTX) and docetaxel and ramucirumab (DTX/RAM) treatment groups, neither in the immune checkpoint inhibitor (ICI)-pretreated group nor in the immune checkpoint inhibitor (ICI)-untreated group. There was a significant difference in overall survival between DTX and DTX/RAM groups in the ICI-untreated group. Patients treated with DTX/RAM have a longer survival time than those treated with DTX in the ICI-pretreated group, although the difference was not significant. MST, median survival time.

Subsequently, the median time-to-treatment failure and the overall survival were compared between the DTX-treated and DTX/RAM-treated groups (Figure 2). The median time-to-treatment failure and the overall survival were significantly longer in cases treated with DTX/RAM than in the DTX-treated group (Figure 2). To evaluate the influence of ICI treatment on the response to DTX/RAM or DTX, the median time-to-treatment failure and the overall survival were compared between DTX-treated and DTX/RAM-treated patients in each ICI-pretreated group and ICI-untreated group. The median time-to-treatment failure was not significantly different between DTX and DTX/RAM subgroups in either ICI-pretreated or ICI-untreated patients (Figure 3). However, the DTX/RAM subgroup showed significantly increased mean survival time compared to the DTX subgroup in the ICI-untreated population. The DTX/RAM subgroup also had a better mean survival time than the DTX subgroup in the ICI-pretreated patients, although the difference was not statistically different (Figure 3).

**Figure 4:**
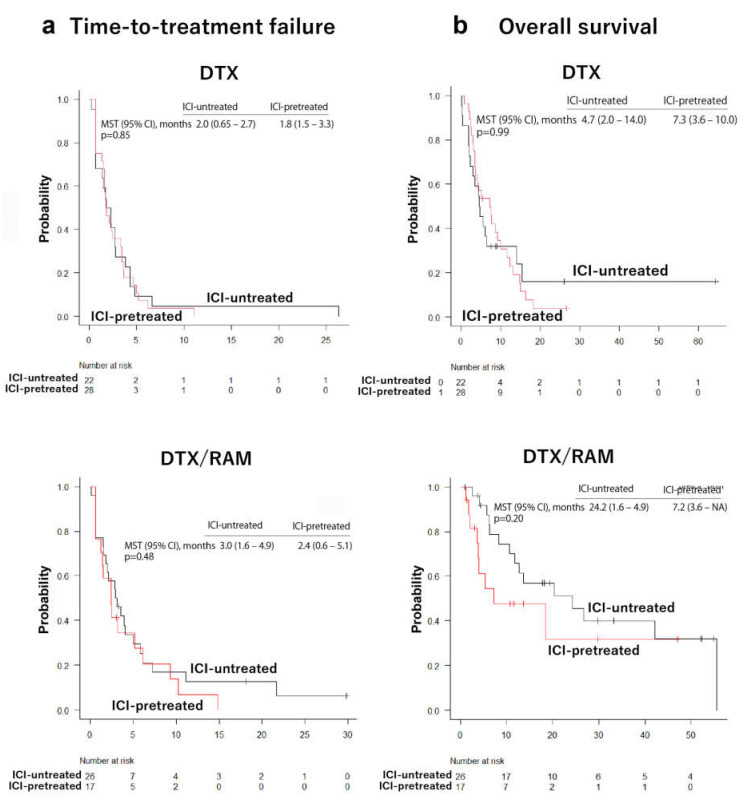
Comparative analysis of immune checkpoint inhibitor-pretreated and -untreated patients in each docetaxel-treated and docetaxel/ramucirumab-treated group. The time-to-treatment failure and overall survival were not significantly different between checkpoint inhibitor-pretreated and –untreated patients in each docetaxel (DTX)-treated and docetaxel/ramucirumab (DTX/RAM)-treated group.

To further clarify the influence of previous ICI treatment on the response to DTX/RAM and DTX, patients from each treatment group were allocated into ICI-pretreated and ICI-untreated subgroups, and the median time-to-treatment failure and the mean survival time were analyzed. The median time-to-treatment failure and the mean survival time were not statistically different between ICI-pretreated and ICI-untreated subgroups in patients treated with DTX or DTX/RAM (Figure 4). These results indicate that neither the therapeutic efficacy of DTX nor that of DTX/RAM is affected by previous ICI treatment.

### 3.4. Univariate Analyses

Univariate analysis was performed to assess whether the time-to-treatment failure or overall survival is correlated with several independent clinical factors. The Eastern Cooperative Oncology Group Performance Status (ECOG PS) and chest CT honeycombing were significant factors in predicting variations in the median time-to-treatment failure, whereas treatment with DTX or DTX/RAM, aging, tumor histological types, and chest CT honeycombing were significant predictors of the median survival time in the univariate analysis (Table 3). Previous ICI treatment was not an important confounding factor in the univariate analysis of the median time-to-treatment failure or median survival time. However, the presence of IPF-like CT honeycombing but no other interstitial lung disease (HR of TTF: 0.83 95% CI 0.40–1.73 *p* = 0.67, HR of OS: 0.80 95% CI 0.31–2.02 *p* = 0.63) was a significant confounding factor (Table 3).

### 3.5. Multivariate Analysis

The multivariate analysis showed that ECOG PS and chest CT honeycombing are significant predictors of variations in the median time-to-treatment failure. Treatment with DTX or DTX/RAM, aging, ECOG PS, and chest CT honeycombing were significant predictors of the mean survival time in the multivariate analysis. Previous ICI treatment was not a significant confounding factor in the multivariate analysis of the median time-to-treatment failure or median survival time. The results were not affected by the histological type of the tumors or by smoking.

### 3.6. Propensity Score Analysis

The clinical outcomes of patients treated with DTX and DTX/RAM were compared after adjusting for age, gender, performance status, smoking status, histology, and driver mutation and the clinical outcomes of the ICI-untreated and ICI-pretreated groups were compared after adjusting for age, gender, smoking status, histology, driver mutation, PD-L1 status, and lung honeycombing. Even after adjustment with propensity scores, treatment with DTX/RAM but no previous history of ICI treatment significantly affected the time-to-treatment failure and overall survival (Table 4).

## 4. Discussion

The use of ICI prior to chemotherapy increases the efficacy of chemotherapy [16,17]. The suppression of bone marrow-derived suppressor cells and regulatory T cells increased immune penetration associated with tumor destruction and the upregulation of death receptors in tumor cells have been suggested as potential mechanisms [17,18]. In addition, the therapeutic effect of ICI appears to persist for several months [19,20]. Increased efficacy of DTX/RAM in patients previously treated with ICI has also been reported [8,9,10,11,13,14]. Similar to previous reports, we found that the objective response rate is higher in patients pretreated with ICI than those without previous ICI treatment [10,13]. However, we found no significant difference in time-to-treatment failure and overall survival between patients with and without a history of ICI treatment [14]. Furthermore, the overall survival after DTX/RAM treatment was significantly better in the ICI-untreated group than in the ICI-pretreated group. Overall, these observations suggest that ICI pretreatment has no impact on the efficacy of DTX or DTX/RAM combination therapy.

The discrepancy of our present investigation with previous studies regarding the better outcome in ICI-untreated patients may be attributed to the inclusion in our current study of an increased number of patients with mutant *epidermal growth factor receptor (EGFR)*-positive lung adenocarcinoma in the DTX/RAM group without previous history of ICI and an increased number of cases of squamous cell carcinoma in the DTX-treated group [21,22,23]. The inclusion of patients with mutant *EGFR*-positive lung adenocarcinoma who became positive for *T790M* and were treated with osimertinib may also be a potential explanation for a better outcome in the ICI-untreated group. We speculated about a superior efficacy of DTX/RAM treatment in *EGFR*-positive than in *EGFR*-negative NSCLC patients, and the reported poor response of squamous cell carcinoma to DTX/RAM treatment may explain the improved survival in the ICI-untreated patients [21,22,23]. Although there is no supporting evidence, the possibility of a shorter half-life of ICI in our ICI-treated group compared to patients from previous studies may also be a potential explanation for the lack of response in the ICI-treated group of the current study. Similar discrepancies between large clinical trials and studies based on real-world clinical data are not uncommon. Unlike the real world of clinical practice, clinical trials are generally conducted in specialized institutions that can recruit a large population of patients with relatively matched backgrounds. This may explain, for example, why our findings in this investigation differed from those reported by Tozuka et al. [14]. The institution where Tozuka et al. conducted their clinical trial is one of Japan’s leading centers for cancer treatment. Therefore, they can enroll patients with less heterogeneous backgrounds in terms of age, performance status, genetic mutations, and underlying diseases [14].

Previous studies have shown that the pre-existence of interstitial lung disease is a poor prognostic factor and that mortality by ICI-related drug-induced lung injury is high in lung cancer patients [24,25]. However, no study has evaluated the influence of pre-existent interstitial lung disease or lung honeycombing on clinical outcomes in NSCLC patients [10,11,14]. In the present study, we found that IPF-like honeycombing findings but no other interstitial lung disease significantly influence the overall survival and the time-to-treatment failure in NSCLC patients. Consistent with our present observation, a previous report has shown that NSCLC patients with interstitial lung disease without honeycombing have no exacerbation of interstitial lung disease after ICI treatment [26]. In the current study, there were patients with CT honeycombing in the ICI-untreated or DTX group, and this may be another explanation for the poor prognosis of patients in this treatment group.

Overall, these observations suggest that the patient background, including gene mutation, interstitial pneumonia, and histological type, must be taken into consideration to assess the therapeutic efficacy of DTX and DTX/RAM in NSCLC patients in the real-world clinical practice. Furthermore, in addition to mutations in the EGFR gene, a previous report has shown that the efficacy of DTX/RAM may also differ depending on the positivity of *Kirsten rat sarcoma viral oncogene homologue (KRAS)* gene mutation [8]. Therefore, studies in a large population that include subjects with different clinical backgrounds and genetic mutations detected using next-generation sequencers should be conducted in the future to confirm these preliminary findings.

The study’s retrospective nature, the small population size, the conduction of the study in a single institution, and the lack of CT studies to evaluate response in some of the patients are the main limitations of the present clinical investigation.

## 5. Conclusions

The present study results show that the combined therapy of DTX and RAM is more effective than DTX irrespective of previous treatment with ICI and that treatment with DTX/RAM, aging, and ECOG PS are significant predictors of patient survival. Based on these findings and in the absence of any risk of hemorrhage, we recommend DTX/RAM combination therapy in NSCLC patients previously treated with ICI. However, studies with a larger population should be performed to confirm these preliminary findings.

## Figures and Tables

**Table 1:** Clinical characteristics of the study population.

Variables		DTX	DTX/RAM	*p*-Values	ICI-Untreated	ICI-Pretreated	*p*-Value
		(*n* = 50)	(*n* = 43)		(*n* = 48)	(*n* = 45)	
Age (years) (%)	<75	32 (64.0)	30 (69.8)	0.66	32 (66.7)	30 (66.7)	1
	≥75	18 (36.0)	13 (30.2)		16 (33.3)	15 (33.3)	
Gender (%)	Female	8 (16.0)	20 (46.5)	0.002	20 (41.7)	8 (17.8)	0.014
	Male	42 (84.0)	23 (53.5)		28 (58.3)	37 (82.2)	
ECOG PS (%)	0	19 (38.0)	26 (60.5)	0.014	22 (45.8)	23 (51.1)	0.686
	1	25 (50.0)	12 (27.9)		21 (43.8)	16 (35.6)	
	2	0 (0.0)	3 (7.0)		2 (4.2)	1 (2.2)	
	3	6 (12.0)	2 (4.7)		3 (6.2)	5 (11.1)	
Smoking status (%)	Non-smoker	5 (10.0)	13 (30.2)	0.018	15 (31.2)	3 (6.7)	0.003
	Smoker	45 (90.0)	30 (69.8)		33 (68.8)	42 (93.3)	
Lung honeycombing (%)	Negative	47 (94.0)	43 (100.0)	0.246	45 (93.8)	45 (100)	0.243
	Positive	3 (6.0)	0 (0.0)		3 (6.2)	0 (0.0)	
Disease stage (%)	II	2 (4.0)	0 (0.0)	0.385	1 (2.1)	1 (2.2)	0.23
	III	16 (32.0)	10 (23.3)		10 (20.8)	16 (35.6)	
	IV	27 (54.0)	24 (55.8)		31 (64.6)	20 (44.4)	
	Recurrence	5 (10.0)	9 (20.9)		6 (12.5)	8 (17.8)	
Histology (%)	Adenocarcinoma	23 (46.0)	36 (83.7)	<0.001	36 (75.0)	23 (51.1)	0.045
	Squamous cell carcinoma	24 (48.0)	5 (11.6)		10 (20.8)	19 (42.2)	
	Large cell carcinoma	0 (0.0)	1 (2.3)		0 (0.0)	1 (2.2)	
	NOS	3 (6.0)	1 (2.3)		2 (4.2)	2 (4.4)	
ALK transfusion (%)	Wild type	34 (68.0)	41 (95.3)	<0.001	40 (83.3)	35 (77.8)	0.424
	Positive	0 (0.0)	1 (2.3)		1 (2.1)	0 (0.0)	
	Not evaluated	16 (32.0)	1 (2.3)		7 (14.6)	10 (22.2)	
EGFR Mutation (%)	Wild type	41 (82.0)	29 (67.4)	0.004	28 (58.3)	42 (93.3)	<0.001
	Exon 19 deletion	5 (10.0)	7 (16.3)		11 (22.9)	1 (2.2)	
	Exon 21 L858R	0 (0.0)	6 (14.0)		6 (12.5)	0 (0.0)	
	Exon 20 insertion	0 (0.0)	1 (2.3)		1 (2.1)	0 (0.0)	
	Not evaluated	4 (8.0)	0 (0.0)		2 (4.2)	2 (4.4)	
EGFR and ALK (%)	Wild type or not evaluated	46 (92.0)	28 (65.1)	0.002	29 (61.7)	45 (97.8)	<0.001
	Mutation positive	4 (8.0)	15 (34.9)		18 (38.3)	1 (2.2)	
PD-L1 status (%)	<1%	16 (32.0)	7 (16.3)	0.162	13 (27.1)	10 (22.2)	0.017
	1–49%	11 (22.0)	7 (16.3)		6 (12.5)	12 (26.7)	
	>50%	8 (16.0)	7 (16.3)		4 (8.3)	11 (24.4)	
	Unknown	15 (30.0)	22 (51.2)		25 (52.1)	12 (26.7)	
Previous ICI treatment (%)	None	22 (44.0)	26 (60.5)	0.128	48 (100.0)	0 (0.0)	<0.001
	Atezolizumab	3 (6.0)	1 (2.3)		0 (0.0)	4 (8.9)	
	Nivolumab	7 (14.0)	4 (9.3)		0 (0.0)	11 (24.4)	
	Pembrolizumab	9 (18.0)	4 (9.3)		0 (0.0)	13 (28.9)	
	CBDCA + nab-PTX + Atezolzumab	0 (0.0)	1 (2.3)		0 (0.0)	1 (2.2)	
	CBDCA + PEM + Pembrolizumab	2 (4.0)	6 (14.0)		0 (0.0)	8 (17.8)	
	CBDCA + nab-PTX + Pembrolizumab	6 (12.0)	1 (2.3)		0 (0.0)	7 (15.6)	
	CBDCA + PTX + Durvalumab	1 (2.0)	0 (0.0)		0 (0.0)	1 (2.2)	
Findings of interstitial pneumonia (%)	Negative	44 (88.0)	40 (93.0)	0.498	41 (85.4)	43 (95.6)	0.16
	Positive	6 (12.0)	3 (7.0)		7 (14.6)	2 (4.4)	

ALK: anaplastic lymphoma kinase; CBDCA: carboplatin; DTX: docetaxel; ECOG PS: Eastern Cooperative Oncology Group Performance Status; EGFR: epidermal growth factor receptor; ICI: immune checkpoint inhibitor; nab-PTX: nab-paclitaxel; NOS: not otherwise specified; PD-L1: programmed death-ligand 1; PEM: pemetrexed; PTX: paclitaxel; and RAM ramucirumab.

**Table 2:** Tumor response rate and disease control rate.

	All Patients		
	Docetaxel	Docetaxel/Ramucirumab	*p*-Value
*n*	50	43	0.017
Complete response (%)	0 (0.0)	1 (2.3)	
Partial response (%)	5 (10.0)	11 (25.6)	
Stable disease (%)	19 (38.0)	13 (30.2)	
Progressive disease (%)	22 (44.0)	9 (20.9)	
Not evaluated (%)	4 (8.0)	9 (20.9)	
Overall response rate (%)	5 (10.9, 95% CI 3.6–23.6)	12 (35.3, 95% CI 19.7–53.5)	
Disease control rate (%)	14 (30.4 95% CI 17.7–45.8)	25 (73.5, 95% CI 55.6–87.1)	
	Docetaxel/ramucirumab-treated group		
	ICI-untreated group	ICI-pretreated group	*p*-Value
*n*	26	17	0.047
Complete response (%)	1 (3.8)	0 (0.0)	
Partial response (%)	3 (11.5)	8 (47.1)	
Stable disease (%)	11 (42.3)	2 (11.8)	
Progressive disease (%)	5 (19.2)	4 (23.5)	
Not evaluated (%)	6 (23.1)	3 (17.6)	
Overall response rate (%)	4 (20.0, 95% CI 5.7–43.7)	8 (57.1, 95% CI 28.9–82.3)	
Disease control rate (%)	15 (75.0, 95% CI 50.9–91.3)	10 (71.4 95% CI 41.9–91.6)	
	Docetaxel-treated group		
	ICI-untreated group	ICI-pretreated group	*p*-Value
*n*	22	28	0.639
Complete response (%)	0 (0.0)	0 (0.0)	
Partial response (%)	1 (4.5)	4 (14.3)	
Stable disease (%)	10 (45.5)	9 (32.1)	
Progressive disease (%)	9 (40.9)	13 (46.4)	
Not evaluated (%)	2 (9.1)	2 (7.1)	
Overall response rate (%)	1 (5.0, 95% CI 0.10–24.9)	4 (15.4, 95% CI 4.4–34.9)	
Disease control rate (%)	11 (55, 95% CI 31.5–76.9)	13 (50, 95% CI 29.9–70.1)	

CI: Confidence interval; ICI: Immune checkpoint inhibitor.

**Table 3:** Univariate and multivariate analyses.

			Time-to-Treatment Failure			
Independent Variables			Univariate Analysis		Multivariate Analysis	
		*n*	Hazard ratio (95% CI)	*p*-Value	Hazard ratio (95% CI)	*p*-Value
Treatment	DTX	50	Ref	0.055	Ref	0.12
	DTX/RAM	43	0.65 (0.42–1.01)		0.66 (0.40–1.11)	
Previous ICI treatment	ICI-untreated	48	Ref	0.32	Ref	0.25
	ICI-pretreated	45	1.23 (0.81–1.88)		1.35 (0.81–2.23)	
Age (years)	<75	62	Ref	0.26	Ref	0.049
	≥75	31	1.29 (0.82–2.03)		1.66 (1.00–2.77)	
ECOG PS	0 or 1	82	Ref	0.0064	Ref	0.052
	≥2	11	2.47 (1.28–4.74)		2.04 (1.00–4.17)	
EGFR and ALK Status	Wild type	74	Ref	0.64	Ref	0.62
	Positive	19	0.88 (0.52–1.48)		1.17 (0.63–2.17)	
Histology	Non-squamous cell	64	Ref	0.2	Ref	0.99
	Squamous cell	29	1.34 (0.85–2.12)		1.00 (0.57–1.75)	
CT honeycombing	negative	90	Ref	0.0045	Ref	0.018
	positive	3	5.77 (1.72–19.39)		5.45 (1.34–22.19)	
			Overall survival			
			Univariate analysis		Multivariate analysis	
		*n*	Hazard ratio (95% CI)	*p*-Value	Hazard ratio (95% CI)	*p*-Value
Treatment	DTX	50	Ref	0.00041	Ref	0.006
	DTX/RAM	43	0.39 (0.23–0.66)		0.38 (0.19–0.76)	
Previous ICI treatment	ICI-untreated	48	Ref	0.055	Ref	0.12
	ICI-pretreated	45	1.61 (0.98–2.64)		1.58 (0.89–2.81)	
Age (years)	<75	62	Ref	0.12	Ref	0.013
	≥75	31	1.49 (0.89–2.49)		2.01 (1.16–3.48)	
ECOG PS	0 or 1	82	Ref	0.0022	Ref	0.033
	≥2	11	3.06 (1.49–6.28)		2.39 (1.08–5.30)	
EGFR and ALK Status	Wild type	74	Ref	0.44	Ref	0.27
	Positive	19	0.79 (0.43–1.43)		1.56 (0.71–3.42)	
Histology	Non-squamous cell	64	Ref	0.018	Ref	0.56
	Squamous cell	29	1.86 (1.11–3.11)		1.20 (0.65–2.24)	
CT honeycombing	Negative	90	Ref	0.0079	Ref	0.045
	Positive	3	5.01 (1.52–16.53)		4.18 (1.03–16.92)	

CI: Confidence interval; DTX: docetaxel; ECOG PS: Eastern Cooperative Oncology Group Performance Status; EGFR: epidermal growth factor receptor; Ref: referent; and CT: computed tomography.

**Table 4:** Hazard ratio adjusted by propensity score.

		*n*	Time-to-Treatment Failure		Overall Survival	
	Treatment		Hazard Ratio (95% CI)	*p*-Value	Hazard Ratio (95% CI)	*p*-Value
Unadjusted	DTX	50	Ref	0.055	Ref	0.00041
	DTX/RAM	43	0.65 (0.42–1.01)		0.39 (0.23–0.66)	
IPTW-weighted	DTX	49	Ref	0.16	Ref	0.00018
	DTX/RAM	41	0.69 (0.41–1.16)		0.37 (0.22–0.62)	
1:1 Matching	DTX	22	Ref	0.22	Ref	0.0034
	DTX/RAM	26	0.69 (0.38–1.25)		0.32 (0.15–0.68)	
Unadjusted	ICI-untreated	48	Ref	0.32	Ref	0.055
	ICI-pretreated	45	1.23 (0.81–1.88)		1.61 (0.98–2.64)	
IPTW-weighted	ICI-untreated	48	Ref	0.35	Ref	0.52
	ICI-pretreated	45	0.68 (0.30–1.53)		1.28 (0.59–2.79)	
1:1 Matching	ICI-untreated	23	Ref	0.5	Ref	0.25
	ICI-pretreated	23	1.23 (0.66–2.27)		1.54 (0.73–3.22)	

CI; confidence intervals, DTX; docetaxel, ICI; immune checkpoint inhibitor, IPTW; inverse probability of treatment weighting and RAM; ramucirumab.

## Data Availability

All data are available under reasonable request from the corresponding author.
